# Crystal structure of *trans*-aqua­(perchlorato-κ*O*)bis­(propane-1,3-di­amine-κ^2^ *N*,*N*′)copper(II) perchlorate

**DOI:** 10.1107/S1600536814023496

**Published:** 2014-10-31

**Authors:** J. Govindaraj, K. Rajkumar, A. S. Ganeshraja, K. Anbalagan, A. SubbiahPandi

**Affiliations:** a Department of Physics, Pachaiyappa’s College for Men, Kanchipuram 631 501, India; b Department of Chemistry, Pondicherry University, Pondicherry 605 014, India; c Department of Physics, Presidency College (Autonomous), Chennai 600 005, India

**Keywords:** crystal structure, propane-1,3-di­amine, copper(II) complex

## Abstract

In the title compound, [Cu(ClO_4_)(C_3_H_10_N_2_)_2_(H_2_O)]ClO_4_, the Cu^II^ atom has a distorted octa­hedral coordination sphere and is coordinated by the N atoms of two propane-1,3-di­amine ligands in the equatorial plane. The axial positions are occupied by a water O atom and an O atom of a disordered perchlorate anion [occupancy ratio 0.631 (9):0.369 (9)]. In the crystal, the various components are linked *via* O—H⋯O, N—H⋯O and C—H⋯O hydrogen bonds, forming sheets lying parallel to (001).

## Abstract 2

In the title compound, the Cu^II^ atom has a distorted octa­hedral coordination sphere coordinated by the N atoms of two propane-1,3-di­amine ligands in the equatorial plane. The axial positions are occupied by a water O atom and an O atom of a disordered perchlorate anion [occupancy ratio 0.631 (9):369 (9)].

## Chemical context   

There have been numerous reports of bis­(propane-1,3-di­amine)­copper(II) complexes, essentially with the copper atom coordinated by the N atoms of the ligands in the equatorial plane of the copper octa­hedral coordination sphere and with two identical O-containing ligands in the axial positions, for example, *trans*-di­aqua­bis­(propane-1,3-di­amine-κ^2^ *N*,*N*′)copper(II) di­thio­nate (Kim *et al.*, 2003 ▶) and bis­[aqua­(1,3-di­amino­propane-κ^2^ *N*,*N*′)]copper(II) difluoride (Emsley *et al.*, 1988 ▶). In order to further develop the coordination chemistry of such copper complexes, we report herein on the synthesis and crystal structure of the title complex, which has two different ligands in the axial positions of the octa­hedral coordination sphere of the copper atom.
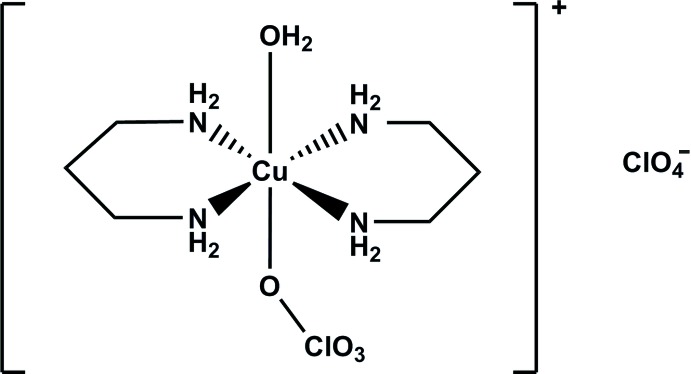


## Structural commentary   

**Figure 1:**
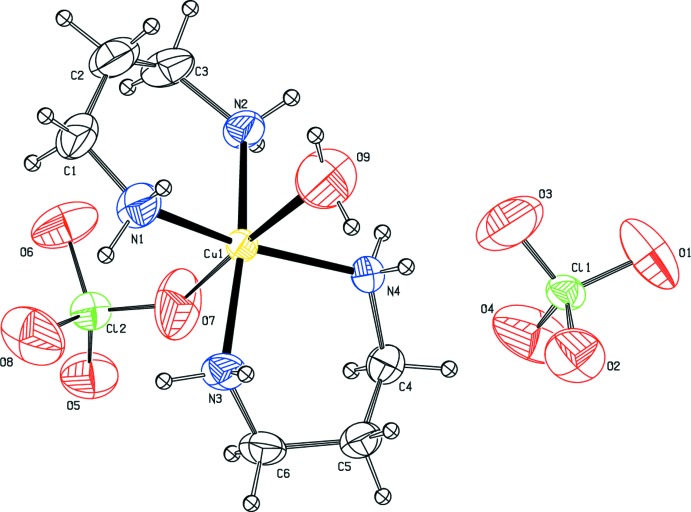
The mol­ecular structure of the title compound, showing the atom labelling. Displacement ellipsoids are drawn at the 30% probability level. The minor components of the disordered coordinating perchlorate anion have been omitted for clarity.

The mol­ecular structure of the title complex is illustrated in Fig. 1 ▶. The Cu^II^ atom has a distorted octa­hedral coordination sphere, reflecting the characteristic Jahn–Teller distortion. It is coordinated by the N atoms of two propane-1,3-di­amine ligands in the equatorial plane with Cu—N bond lengths varying between 2.003 (4)–2.023 (3) Å. The axial positions are occupied by the water O9 atom and by atom O7 of a disordered perchlorate anion [occupancy ratio 0.631 (9):0.369 (9)], with Cu—O bond lengths of 2.585 (6) and 2.680 (10) Å, respectively.

## Supra­molecular features   

**Figure 2:**
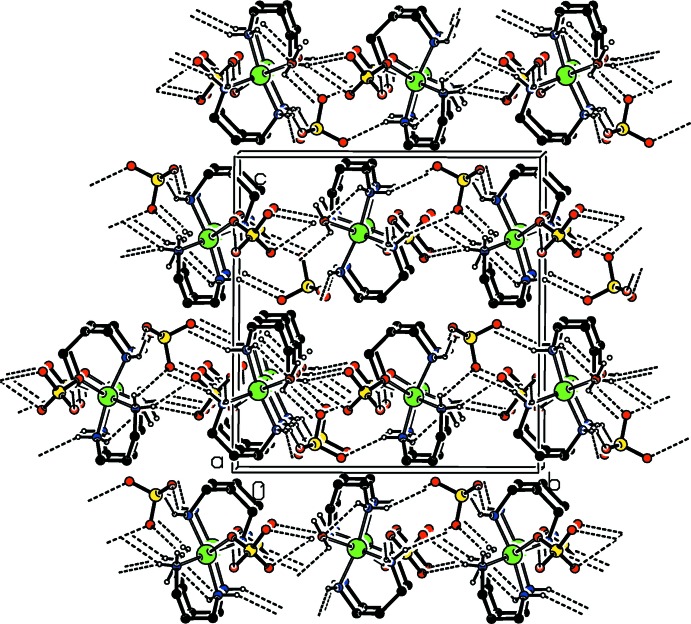
A view along the *a* axis of the crystal structure of the title compound. O—H⋯O and N—H⋯O hydrogen bonds are shown as dashed lines (see Table 1 ▶ for details; the minor components of the disordered coordinating perchlorate anion and the C-bound H atoms have been omitted for clarity)

In the crystal, the various components are linked *via* O—H⋯O, N—H⋯O and C—H⋯O hydrogen bonds forming sheets lying parallel to (001); see Table 1 ▶ and Fig. 2 ▶.

## Synthesis and crystallization   

The complex was prepared by mixing copper(II) perchlorate hexa­hydrate with 1,3-di­amino­propane in a (1:2) molar ratio. Cu(ClO_4_)_2_·6H_2_O (3.7 g, 1 *M*) was dissolved in 15 ml of warm water. After an hour, about 10 ml of an ethanol solution of 1,3-di­amino­propane (1.48 g, 2*M*) was added dropwise with continuous stirring. This solution was then filtered to remove any impurities and the solution was kept over P_2_O_5_ in a desiccator. Finally, violet–purple-coloured crystals suitable for X-ray diffraction analysis were harvested and washed repeatedly with cold water (yield 70%).

## Refinement   

Crystal data, data collection and structure refinement details are summarized in Table 2 ▶. The water H atoms were located in a difference Fourier map and refined with a distance restraint, O—H = 0.90 (2) Å, and with *U*_iso_(H) = 1.5*U*_eq_(O). The N -and C-bound H atoms were positioned geometrically and allowed to ride on their parent atoms, with N—H = 0.90 and C—H = 0.97 Å, and with *U*_iso_(H) = 1.2*U*_eq_(N,C). The disordered coordinating perchlorate anion, involving atom Cl2, was refined with an occupancy ratio of 0.631 (9):0.369 (9).

## Supplementary Material

Crystal structure: contains datablock(s) global, I. DOI: 10.1107/S1600536814023496/su2799sup1.cif

Structure factors: contains datablock(s) I. DOI: 10.1107/S1600536814023496/su2799Isup2.hkl

CCDC reference: 1031014

Additional supporting information:  crystallographic information; 3D view; checkCIF report

## Figures and Tables

**Table 1:** Hydrogen-bond geometry (, )

*D*H*A*	*D*H	H*A*	*D* *A*	*D*H*A*
O9H9*B*O3^i^	0.90(2)	2.38(11)	2.917(9)	118(9)
N1H1*C*O1	0.90	2.22	3.040(7)	151
N1H1*D*O1^ii^	0.90	2.69	3.511(8)	151
N1H1*D*O9	0.90	2.41	2.927(8)	117
N2H2*C*O5^iii^	0.90	2.58	3.443(12)	162
N2H2*C*O8^iii^	0.90	2.42	3.183(10)	143
N2H2*C*O5^iii^	0.90	2.39	3.23(3)	156
N2H2*D*O3^iii^	0.90	2.70	3.449(11)	141
N3H3*C*O6^iv^	0.90	2.15	3.014(9)	160
N3H3*C*O7^iv^	0.90	2.36	3.246(17)	169
N3H3*D*O3	0.90	2.28	3.137(8)	160
N4H4*C*O2^iii^	0.90	2.35	3.127(6)	144
N4H4*D*O4^i^	0.90	2.45	3.223(7)	145
C4H4*A*O5^v^	0.97	2.47	3.264(14)	139
C5H5*B*O4^i^	0.97	2.63	3.368(7)	133

Symmetry codes: (i) 
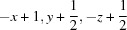
; (ii) 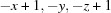; (iii) 
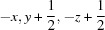
; (iv) 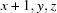; (v) 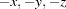.

**Table 2:** Experimental details

Crystal data	
Chemical formula	[Cu(ClO_4_)(C_3_H_10_N_2_)_2_(H_2_O)]ClO_4_
*M*_r_	428.72
Crystal system, space group	Monoclinic, *P*2_1_/*c*
Temperature (K)	293
*a*, *b*, *c* ()	7.8563(4), 14.2936(6), 14.8769(7)
()	100.022(5)
*V* (^3^)	1645.11(13)
*Z*	4
Radiation type	Mo *K*
(mm^1^)	1.70
Crystal size (mm)	0.30 0.30 0.25

Data collection	
Diffractometer	Oxford Diffraction Xcalibur with an Eos detector
Absorption correction	Multi-scan (*CrysAlis PRO*; Oxford Diffraction, 2009 ▶)
*T*_min_, *T*_max_	0.607, 0.654
No. of measured, independent and observed [*I* > 2(*I*)] reflections	7573, 2900, 2353
*R*_int_	0.032
(sin /)_max_ (^1^)	0.595

Refinement	
*R*[*F* ^2^ > 2(*F* ^2^)], *wR*(*F* ^2^), *S*	0.046, 0.127, 1.06
No. of reflections	2900
No. of parameters	242
No. of restraints	109
H-atom treatment	H atoms treated by a mixture of independent and constrained refinement
_max_, _min_ (e ^3^)	0.60, 0.42

Computer programs: *CrysAlis CCD* and *CrysAlis RED* (Oxford Diffraction, 2009 ▶), *SHELXS97* and *SHELXL97* (Sheldrick, 2008 ▶) and *PLATON* (Spek, 2009 ▶).

## supplementary crystallographic information

### Crystal data

**Table:**

[Cu(ClO_4_)(C_3_H_10_N_2_)_2_(H_2_O)]ClO_4_	*F*(000) = 884
*M**_r_* = 428.72	*D*_x_ = 1.731 Mg m^−^^3^
Monoclinic, *P*2_1_/*c*	Mo *K*α radiation, λ = 0.71073 Å
Hall symbol: -P 2ybc	Cell parameters from 2353 reflections
*a* = 7.8563 (4) Å	θ = 3.8–25.0°
*b* = 14.2936 (6) Å	µ = 1.70 mm^−^^1^
*c* = 14.8769 (7) Å	*T* = 293 K
β = 100.022 (5)°	Block, violet-purple
*V* = 1645.11 (13) Å^3^	0.30 × 0.30 × 0.25 mm
*Z* = 4	

### Data collection

**Table:**

Oxford diffraction Xcalibur diffractometer with an Eos detector	2900 independent reflections
Radiation source: fine-focus sealed tube	2353 reflections with *I* > 2σ(*I*)
Graphite monochromator	*R*_int_ = 0.032
ω and φ scans	θ_max_ = 25.0°, θ_min_ = 3.8°
Absorption correction: multi-scan (*CrysAlis PRO*; Oxford Diffraction, 2009)	*h* = −9→9
*T*_min_ = 0.607, *T*_max_ = 0.654	*k* = −16→16
7573 measured reflections	*l* = −17→16

### Refinement

**Table:**

Refinement on *F*^2^	Primary atom site location: structure-invariant direct methods
Least-squares matrix: full	Secondary atom site location: difference Fourier map
*R*[*F*^2^ > 2σ(*F*^2^)] = 0.046	Hydrogen site location: inferred from neighbouring sites
*wR*(*F*^2^) = 0.127	H atoms treated by a mixture of independent and constrained refinement
*S* = 1.06	*w* = 1/[σ^2^(*F*_o_^2^) + (0.0667*P*)^2^ + 1.2858*P*] where *P* = (*F*_o_^2^ + 2*F*_c_^2^)/3
2900 reflections	(Δ/σ)_max_ = 0.002
242 parameters	Δρ_max_ = 0.60 e Å^−^^3^
109 restraints	Δρ_min_ = −0.42 e Å^−^^3^

### Special details

**Table:**

Geometry. All e.s.d.'s (except the e.s.d. in the dihedral angle between two l.s. planes) are estimated using the full covariance matrix. The cell e.s.d.'s are taken into account individually in the estimation of e.s.d.'s in distances, angles and torsion angles; correlations between e.s.d.'s in cell parameters are only used when they are defined by crystal symmetry. An approximate (isotropic) treatment of cell e.s.d.'s is used for estimating e.s.d.'s involving l.s. planes.
Refinement. Refinement of *F*^2^ against ALL reflections. The weighted *R*-factor *wR* and goodness of fit *S* are based on *F*^2^, conventional *R*-factors *R* are based on *F*, with *F* set to zero for negative *F*^2^. The threshold expression of *F*^2^ > σ(*F*^2^) is used only for calculating *R*-factors(gt) *etc*. and is not relevant to the choice of reflections for refinement. *R*-factors based on *F*^2^ are statistically about twice as large as those based on *F*, and *R*- factors based on ALL data will be even larger.

### Fractional atomic coordinates and isotropic or equivalent isotropic displacement parameters (Å^2^)

**Table:**

	*x*	*y*	*z*	*U*_iso_*/*U*_eq_	Occ. (<1)
C1	0.2060 (10)	0.0648 (5)	0.4655 (4)	0.0879 (19)	
H1A	0.2841	0.0599	0.5234	0.105*	
H1B	0.1231	0.0142	0.4630	0.105*	
C2	0.1130 (10)	0.1523 (5)	0.4648 (4)	0.099 (2)	
H2A	0.0517	0.1508	0.5160	0.119*	
H2B	0.1985	0.2017	0.4771	0.119*	
C3	−0.0098 (8)	0.1800 (5)	0.3854 (4)	0.0846 (17)	
H3A	−0.0506	0.2423	0.3965	0.101*	
H3B	−0.1084	0.1383	0.3802	0.101*	
C4	0.1631 (8)	0.0875 (3)	0.0573 (3)	0.0667 (14)	
H4A	0.1641	0.1259	0.0037	0.080*	
H4B	0.0489	0.0602	0.0524	0.080*	
C5	0.2934 (7)	0.0105 (3)	0.0589 (3)	0.0659 (13)	
H5A	0.2758	−0.0197	−0.0004	0.079*	
H5B	0.4083	0.0376	0.0693	0.079*	
C6	0.2841 (8)	−0.0610 (3)	0.1296 (3)	0.0700 (14)	
H6A	0.1671	−0.0851	0.1218	0.084*	
H6B	0.3597	−0.1126	0.1208	0.084*	
N1	0.3041 (7)	0.0504 (4)	0.3943 (3)	0.0860 (15)	
H1C	0.3285	−0.0111	0.3940	0.103*	
H1D	0.4054	0.0803	0.4113	0.103*	
N2	0.0484 (6)	0.1812 (3)	0.2978 (3)	0.0705 (12)	
H2C	0.0951	0.2380	0.2925	0.085*	
H2D	−0.0471	0.1780	0.2546	0.085*	
N3	0.3333 (6)	−0.0259 (3)	0.2235 (3)	0.0678 (11)	
H3C	0.4473	−0.0136	0.2325	0.081*	
H3D	0.3180	−0.0730	0.2614	0.081*	
N4	0.1967 (6)	0.1474 (2)	0.1393 (2)	0.0573 (10)	
H4C	0.1117	0.1902	0.1342	0.069*	
H4D	0.2958	0.1786	0.1382	0.069*	
O1	0.3452 (8)	−0.1512 (3)	0.4593 (5)	0.149 (2)	
O2	0.1810 (6)	−0.2818 (4)	0.4266 (4)	0.1161 (16)	
O3	0.3230 (12)	−0.2194 (6)	0.3219 (5)	0.185 (3)	
O4	0.4749 (7)	−0.2926 (4)	0.4506 (5)	0.156 (3)	
Cl1	0.33528 (15)	−0.23745 (7)	0.41570 (8)	0.0557 (3)	
Cl2	−0.18163 (15)	−0.06425 (9)	0.24310 (7)	0.0576 (3)	
Cu1	0.21563 (6)	0.08711 (3)	0.26368 (3)	0.0411 (2)	
O5	−0.2890 (12)	−0.1162 (7)	0.1754 (6)	0.102 (3)	0.631 (9)
O6	−0.2845 (9)	−0.0126 (8)	0.2958 (6)	0.109 (3)	0.631 (9)
O7	−0.0828 (12)	−0.0013 (7)	0.2023 (6)	0.133 (4)	0.631 (9)
O8	−0.0665 (16)	−0.1226 (7)	0.3013 (6)	0.142 (4)	0.631 (9)
O5'	−0.188 (3)	−0.1082 (17)	0.1585 (9)	0.156 (9)	0.369 (9)
O6'	−0.0084 (12)	−0.0497 (11)	0.2830 (12)	0.117 (5)	0.369 (9)
O7'	−0.2628 (19)	0.0227 (8)	0.2243 (14)	0.131 (6)	0.369 (9)
O8'	−0.258 (2)	−0.1212 (13)	0.2983 (10)	0.138 (6)	0.369 (9)
O9	0.4814 (7)	0.1951 (5)	0.3062 (4)	0.133 (2)	
H9B	0.557 (10)	0.173 (8)	0.272 (6)	0.200*	
H9A	0.536 (11)	0.222 (7)	0.357 (4)	0.200*	

### Atomic displacement parameters (Å^2^)

**Table:**

	*U*^11^	*U*^22^	*U*^33^	*U*^12^	*U*^13^	*U*^23^
C1	0.121 (5)	0.097 (4)	0.048 (3)	0.019 (4)	0.022 (3)	0.011 (3)
C2	0.141 (6)	0.105 (5)	0.058 (3)	0.035 (5)	0.032 (4)	−0.006 (3)
C3	0.081 (4)	0.101 (4)	0.076 (4)	0.024 (3)	0.026 (3)	−0.019 (3)
C4	0.092 (4)	0.062 (3)	0.045 (2)	0.005 (3)	0.009 (3)	0.003 (2)
C5	0.073 (3)	0.067 (3)	0.064 (3)	−0.004 (3)	0.028 (3)	−0.013 (2)
C6	0.081 (4)	0.054 (3)	0.076 (3)	0.015 (3)	0.017 (3)	−0.014 (2)
N1	0.115 (4)	0.086 (3)	0.056 (2)	0.043 (3)	0.013 (3)	0.019 (2)
N2	0.088 (3)	0.066 (3)	0.063 (2)	0.036 (2)	0.028 (2)	0.0098 (19)
N3	0.078 (3)	0.060 (2)	0.064 (2)	0.031 (2)	0.010 (2)	0.0014 (19)
N4	0.080 (3)	0.045 (2)	0.049 (2)	0.0049 (19)	0.0173 (19)	0.0063 (15)
O1	0.138 (5)	0.072 (3)	0.221 (6)	0.001 (3)	−0.014 (4)	−0.046 (4)
O2	0.078 (3)	0.114 (3)	0.163 (5)	−0.024 (3)	0.039 (3)	0.001 (3)
O3	0.245 (9)	0.197 (7)	0.139 (5)	0.046 (6)	0.107 (6)	0.062 (5)
O4	0.080 (3)	0.107 (4)	0.276 (8)	0.042 (3)	0.021 (4)	0.059 (5)
Cl1	0.0524 (6)	0.0451 (6)	0.0733 (7)	0.0054 (5)	0.0212 (6)	0.0074 (5)
Cl2	0.0526 (6)	0.0603 (7)	0.0599 (7)	−0.0013 (5)	0.0092 (5)	−0.0059 (5)
Cu1	0.0435 (3)	0.0393 (3)	0.0415 (3)	0.0075 (2)	0.0096 (2)	0.00435 (19)
O5	0.104 (6)	0.099 (5)	0.097 (6)	−0.017 (5)	0.002 (5)	−0.030 (5)
O6	0.069 (4)	0.162 (9)	0.098 (5)	0.007 (5)	0.019 (4)	−0.049 (6)
O7	0.109 (7)	0.167 (8)	0.127 (7)	−0.064 (6)	0.037 (6)	0.023 (6)
O8	0.185 (10)	0.101 (6)	0.117 (6)	0.043 (7)	−0.040 (7)	0.016 (5)
O5'	0.23 (2)	0.172 (15)	0.059 (8)	0.010 (18)	0.020 (12)	−0.023 (9)
O6'	0.045 (6)	0.115 (11)	0.184 (14)	−0.002 (7)	−0.002 (7)	0.008 (10)
O7'	0.108 (11)	0.064 (7)	0.203 (16)	0.002 (7)	−0.022 (12)	−0.012 (9)
O8'	0.128 (12)	0.182 (15)	0.109 (10)	−0.019 (12)	0.036 (9)	0.044 (10)
O9	0.118 (4)	0.150 (5)	0.133 (5)	−0.054 (4)	0.024 (4)	−0.030 (4)

### Geometric parameters (Å, º)

**Table:**

C1—N1	1.429 (7)	N2—H2C	0.9000
C1—C2	1.447 (8)	N2—H2D	0.9000
C1—H1A	0.9700	N3—Cu1	2.003 (4)
C1—H1B	0.9700	N3—H3C	0.9000
C2—C3	1.445 (9)	N3—H3D	0.9000
C2—H2A	0.9700	N4—Cu1	2.023 (3)
C2—H2B	0.9700	N4—H4C	0.9000
C3—N2	1.454 (6)	N4—H4D	0.9000
C3—H3A	0.9700	O1—Cl1	1.388 (5)
C3—H3B	0.9700	O2—Cl1	1.402 (4)
C4—N4	1.476 (6)	O3—Cl1	1.406 (6)
C4—C5	1.500 (7)	O4—Cl1	1.377 (5)
C4—H4A	0.9700	Cl2—O8'	1.368 (11)
C4—H4B	0.9700	Cl2—O7	1.395 (7)
C5—C6	1.478 (7)	Cl2—O5'	1.399 (12)
C5—H5A	0.9700	Cl2—O6'	1.402 (9)
C5—H5B	0.9700	Cl2—O7'	1.403 (10)
C6—N3	1.471 (6)	Cl2—O5	1.408 (7)
C6—H6A	0.9700	Cl2—O8	1.411 (7)
C6—H6B	0.9700	Cl2—O6	1.425 (6)
N1—Cu1	2.015 (4)	O9—H9B	0.90 (2)
N1—H1C	0.9000	O9—H9A	0.89 (2)
N1—H1D	0.9000	Cu1—09	2.585 (6)
N2—Cu1	2.006 (4)	Cu1—O7	2.680 (1)
			
N1—C1—C2	117.2 (5)	C6—N3—H3D	107.3
N1—C1—H1A	108.0	Cu1—N3—H3D	107.3
C2—C1—H1A	108.0	H3C—N3—H3D	106.9
N1—C1—H1B	108.0	C4—N4—Cu1	118.9 (3)
C2—C1—H1B	108.0	C4—N4—H4C	107.6
H1A—C1—H1B	107.3	Cu1—N4—H4C	107.6
C3—C2—C1	120.5 (5)	C4—N4—H4D	107.6
C3—C2—H2A	107.2	Cu1—N4—H4D	107.6
C1—C2—H2A	107.2	H4C—N4—H4D	107.0
C3—C2—H2B	107.2	O4—Cl1—O1	110.8 (4)
C1—C2—H2B	107.2	O4—Cl1—O2	110.2 (3)
H2A—C2—H2B	106.8	O1—Cl1—O2	109.1 (4)
C2—C3—N2	117.8 (5)	O4—Cl1—O3	113.1 (5)
C2—C3—H3A	107.9	O1—Cl1—O3	106.8 (5)
N2—C3—H3A	107.9	O2—Cl1—O3	106.6 (5)
C2—C3—H3B	107.9	O8'—Cl2—O7	169.0 (9)
N2—C3—H3B	107.9	O8'—Cl2—O5'	108.7 (11)
H3A—C3—H3B	107.2	O7—Cl2—O5'	80.5 (10)
N4—C4—C5	112.9 (4)	O8'—Cl2—O6'	109.2 (10)
N4—C4—H4A	109.0	O7—Cl2—O6'	61.0 (7)
C5—C4—H4A	109.0	O5'—Cl2—O6'	109.2 (10)
N4—C4—H4B	109.0	O8'—Cl2—O7'	114.5 (10)
C5—C4—H4B	109.0	O7—Cl2—O7'	67.0 (8)
H4A—C4—H4B	107.8	O5'—Cl2—O7'	105.9 (13)
C6—C5—C4	113.7 (4)	O6'—Cl2—O7'	109.1 (8)
C6—C5—H5A	108.8	O8'—Cl2—O5	81.0 (9)
C4—C5—H5A	108.8	O7—Cl2—O5	109.8 (6)
C6—C5—H5B	108.8	O5'—Cl2—O5	36.4 (9)
C4—C5—H5B	108.8	O6'—Cl2—O5	142.9 (7)
H5A—C5—H5B	107.7	O7'—Cl2—O5	97.5 (8)
N3—C6—C5	113.7 (4)	O8'—Cl2—O8	65.3 (8)
N3—C6—H6A	108.8	O7—Cl2—O8	107.6 (6)
C5—C6—H6A	108.8	O5'—Cl2—O8	101.9 (11)
N3—C6—H6B	108.8	O6'—Cl2—O8	50.0 (7)
C5—C6—H6B	108.8	O7'—Cl2—O8	150.1 (8)
H6A—C6—H6B	107.7	O5—Cl2—O8	111.6 (6)
C1—N1—Cu1	122.5 (4)	O8'—Cl2—O6	68.1 (8)
C1—N1—H1C	106.7	O7—Cl2—O6	108.5 (6)
Cu1—N1—H1C	106.7	O5'—Cl2—O6	142.2 (11)
C1—N1—H1D	106.7	O6'—Cl2—O6	106.9 (7)
Cu1—N1—H1D	106.7	O7'—Cl2—O6	50.9 (8)
H1C—N1—H1D	106.6	O5—Cl2—O6	109.9 (5)
C3—N2—Cu1	122.8 (3)	O8—Cl2—O6	109.5 (6)
C3—N2—H2C	106.6	N3—Cu1—N2	166.5 (2)
Cu1—N2—H2C	106.6	N3—Cu1—N1	88.76 (17)
C3—N2—H2D	106.6	N2—Cu1—N1	93.53 (17)
Cu1—N2—H2D	106.6	N3—Cu1—N4	91.99 (15)
H2C—N2—H2D	106.6	N2—Cu1—N4	89.94 (15)
C6—N3—Cu1	120.0 (3)	N1—Cu1—N4	161.9 (2)
C6—N3—H3C	107.3	H9B—O9—H9A	111 (3)
Cu1—N3—H3C	107.3		
			
N1—C1—C2—C3	56.0 (10)	C6—N3—Cu1—N4	−35.4 (4)
C1—C2—C3—N2	−53.1 (10)	C3—N2—Cu1—N3	79.8 (9)
N4—C4—C5—C6	67.7 (6)	C3—N2—Cu1—N1	−19.6 (5)
C4—C5—C6—N3	−66.7 (6)	C3—N2—Cu1—N4	178.1 (5)
C2—C1—N1—Cu1	−41.1 (9)	C1—N1—Cu1—N3	−144.4 (5)
C2—C3—N2—Cu1	35.8 (8)	C1—N1—Cu1—N2	22.4 (5)
C5—C6—N3—Cu1	54.2 (6)	C1—N1—Cu1—N4	123.0 (6)
C5—C4—N4—Cu1	−55.5 (5)	C4—N4—Cu1—N3	36.2 (4)
C6—N3—Cu1—N2	62.7 (8)	C4—N4—Cu1—N2	−130.4 (4)
C6—N3—Cu1—N1	162.7 (4)	C4—N4—Cu1—N1	128.3 (6)

### Hydrogen-bond geometry (Å, º)

**Table:**

*D*—H···*A*	*D*—H	H···*A*	*D*···*A*	*D*—H···*A*
O9—H9*B*···O3^i^	0.90 (2)	2.38 (11)	2.917 (9)	118 (9)
N1—H1*C*···O1	0.90	2.22	3.040 (7)	151
N1—H1*D*···O1^ii^	0.90	2.69	3.511 (8)	151
N1—H1*D*···O9	0.90	2.41	2.927 (8)	117
N2—H2*C*···O5^iii^	0.90	2.58	3.443 (12)	162
N2—H2*C*···O8^iii^	0.90	2.42	3.183 (10)	143
N2—H2*C*···O5′^iii^	0.90	2.39	3.23 (3)	156
N2—H2*D*···O3^iii^	0.90	2.70	3.449 (11)	141
N3—H3*C*···O6^iv^	0.90	2.15	3.014 (9)	160
N3—H3*C*···O7′^iv^	0.90	2.36	3.246 (17)	169
N3—H3*D*···O3	0.90	2.28	3.137 (8)	160
N4—H4*C*···O2^iii^	0.90	2.35	3.127 (6)	144
N4—H4*D*···O4^i^	0.90	2.45	3.223 (7)	145
C4—H4*A*···O5′^v^	0.97	2.47	3.264 (14)	139
C5—H5*B*···O4^i^	0.97	2.63	3.368 (7)	133

Symmetry codes: (i) −*x*+1, *y*+1/2, −*z*+1/2; (ii) −*x*+1, −*y*, −*z*+1; (iii) −*x*, *y*+1/2, −*z*+1/2; (iv) *x*+1, *y*, *z*; (v) −*x*, −*y*, −*z*.
